# High CT Attenuation Values Relative to the Brainstem Predict Fungal Hyphae Within the Sinus

**DOI:** 10.3389/fsurg.2022.876340

**Published:** 2022-06-16

**Authors:** Shu Kikuta, Bing Han, Shintaro Yoshihara, Hironobu Nishijima, Kenji Kondo, Tatsuya Yamasoba

**Affiliations:** Department of Otolaryngology, Graduate School of Medicine, University of Tokyo, Tokyo, Japan

**Keywords:** sinus fungal ball, unilateral chronic sinusitis, computed tomography, diagnosis, brainstem

## Abstract

**Objectives:**

There is currently no established objective diagnostic indicator for the differentiation of sinus fungal ball (SFB) from unilateral nonfungal chronic sinusitis (UCRS). This study evaluated whether computed tomography (CT) attenuation values relative to those of the brainstem (relative CT number) are useful for differentiating SFB from UCRS.

**Materials and Methods:**

Consecutive patients who were pathologically diagnosed with SFB or UCRS between 2013 and 2021 were retrospectively identified. The relative CT numbers of region of interest (ROIs) within the sinuses were compared between the two patient groups. Factors with predictive power for differentiating SFBs from UCRSs were identified by uni/multivariable logistic regression analyses.

**Results:**

One hundred and eighty-three patients with unilateral chronic sinusitis were finally analyzed (SFB, 86 cases; UCRS, 97 cases). Regardless of the presence or absence of calcified lesions, the relative CT numbers in SFB were significantly higher than those in UCRS. ROIs showing high relative CT numbers were those where fungal hyphae were present. In the uni/multivariable logistic regression analysis, age (*p* < 0.001), relative CT number (*p* < 0.001), and calcification (*p* = 0.002) had predictive value for distinguishing SFB from UCRS. Within those cases not showing calcification, age (*p* = 0.004) and relative CT number (*p* < 0.001) were predictive factors for differentiating SFB from UCRS. A relative CT number >1.5 was significantly associated with SFB (sensitivity, 70%; specificity, 91%), with a significantly larger area under the receiver operating characteristics curve than age.

**Conclusions:**

High relative CT numbers within the sinus are strongly associated with the presence of fungal hyphae, and measurement of relative CT number is a powerful adjunctive diagnostic method for distinguishing between SFB and UCRS.

## Introduction

The sinus fungal ball (SFB) is the most frequent form of non-invasive fungal rhinosinusitis and is characterized by accumulation of fungal hyphae within the sinus cavity without microscopic evidence of tissue invasion. SFB has appeared with increasing frequency in recent years (1–3) and was reported in about 10% of patients requiring endoscopic sinus surgery (4, 5). SFB can be eradicated completely with surgical treatment, which usually achieves good outcomes including low recurrence and high symptom-free rates (5–7). However, as host immunity deteriorates, SFB may shift to an invasive form with extension to the sinus mucosa, bone, or blood vessels (8). Therefore, prompt and accurate diagnosis of SFB is critical to avoid unnecessary medical therapy and treatment delays.

Among the currently available diagnostic tools, computed tomography (CT) is the optimal imaging technique for the preoperative detection of SFB. One of the most common CT findings is the presence of intralesional hyperdensity with metal-like calcified materials, and this intralesional hyperdensity is known to be a highly predictive radiological parameter for SFB (4, 9). However, this finding involves a subjective assessment, which may lead to variability in results across evaluators. In addition, calcified lesions are often not detected on CT scans, with 18%–50% of patients with SFB not showing them (1, 2, 10–12). Therefore, in cases where calcification does not occur, it may be difficult to distinguish SFB from unilateral chronic rhinosinusitis (UCRS) on the basis of presurgical CT findings (13). Thus, it would be very useful to have a new objective indicator for the detection of fungal hyphae.

The CT attenuation value represents the radiodensity of a material in respect to water and is expressed in Hounsfield units (14). We previously reported on the utility of CT attenuation values of individual sinonasal tumors relative to those of the brainstem (defined by relative CT number) as a possible tool for differentiating individual tumor types (15). We hypothesized that because of the presence of dense matted fungal hyphae, SFB would show high relative CT numbers in comparison with those of the brainstem. The purpose of this study was to investigate whether relative CT number can be used as an objective adjunct diagnostic indicator for diagnosing SFB, and to verify whether relative CT numbers can predict the presence of fungal hyphae even in patients without calcification. We thus performed a retrospective cohort study on patients with SFB and patients with UCRS.

## Materials and Methods

### Ethical Approval

The current study was approved by the Institutional Review Board of the University of Tokyo Hospital (approval no. 2487). All procedures performed in studies involving human participants were in accordance with the ethical standards of the institutional and/or national committee, and with the Helsinki Declaration and its later amendments or comparable ethical standards.

### Patient Selection

Consecutive patients who were pathologically diagnosed with SFB or UCRS at the University of Tokyo Hospital between 2013 and 2021 were identified by reviewing the medical database. Recurrent cases and cases in which regions of interest (ROIs) could not be accurately defined on film CT or contrast-enhanced CT were excluded from the analysis. In some cases, MRI was performed to assess internal features. The following demographic and clinical information was also recorded: age, sex, side, disease duration, nasal symptom (NO, nasal obstruction; NB, nasal bleeding; ND, nasal discharge; PND, postnasal discharge; HD, headache), body mass index (BMI), and Brinkman index (BI). Disease duration was defined as the interval from the onset of symptoms to the date of the CT acquisition and was recorded as months. The BI is the number of cigarettes smoked per day multiplied by the number of years of smoking.

### Analysis of CT Scans

The following lesion characteristics were analyzed on unenhanced CT: the size of the region of interest (ROI) within the sinus (sinus ROI, mm^2^), the size of the brainstem ROI (mm^2^), CT attenuation values (HU), affected sinus, bony changes, and presence of complete opacification. At least six image slices (for both SFBs and UCRSs) were selected from axial unenhanced CT images, and the mean lesion CT attenuation values were measured using ROI settings*.* At least six slices with the same slice thickness were selected for measurement of the brainstem values (15). Surrounding bony structures and aerated areas inside the sinuses were excluded from the ROI measurements. It was difficult to accurately distinguish the mucus region from mucosal thickening. Therefore, the sinus ROIs were defined as the sinus areas located at least a few millimeters medial to the sinus bony wall, while taking into account the thickness of the sinus mucosal lining. The full sinus ROIs were defined as the sinus regions along the sub-sinus ostium when the sinuses were completely filled with soft tissue density material (presence of complete opacification; SFB, 50 cases; UCRS, 45 cases, Table 2).

**Table 1:** Demographic information of the patients with SFB and UCRS (*N* = 183).

	Age (years)	Sex	Side	Disease duration (months)	Nasal symptom					BMI	BI
					NO	NB	ND	PND	HD		
SFB (*N* = 86)	60.3 ± 12.69	M;32 (33%) F;54 (67%)	R;47 (55%) L;39 (45%)	12.2 ± 20.8	13 (15%)	6 (7%)	29 (34%)	24 (28%)	13 (15%)	22.7 ± 4.0	192.8 ± 439.2
UCRS (*N* = 97)	45.4 ± 15.64	M;56 (58%) F;41 (42%)	R;53 (55%) L;44 (45%)	11.2 ± 23.9	20 (21%)	4 (4%)	55 (57%)	27 (28%)	7 (7%)	23.1 ± 3.7	160.2 ± 258.4

*SFB, sinus fungal ball; UCRS, unilateral nonfungal chronic rhinosinusitis; M, male; F, female; R, right; L, left; NO, nasal obstruction; NB, nasal bleeding; ND, nasal discharge; PND, postnasal discharge; HD, headache; BMI, body mass index; BI, Brinkman index.*

**Table 2:** CT findings for SFB and UCRS (*N* = 183).

	Affected sinus				Presence of calcification	Presence of bone thickness	Presence of bone erosion	Presence of complete opacification
	MS	ES	FS	SS				
SFB (*N* = 86)	72 (84%)	35 (41%)	7 (8%)	14 (16%)	64 (74%)	33 (38%)	11 (13%)	50 (58%)
UCRS (*N* = 97)	92 (95%)	56 (58%)	26 (27%)	10 (10%)	18 (19%)	41 (42%)	12 (12%)	45 (46%)

*SFB, sinus fungal ball; UCRS, unilateral nonfungal chronic rhinosinusitis; MS, maxillary sinus; ES, ethmoidal sinus; FS, frontal sinus; SS, sphenoid sinus.*

The fungal ROIs were defined as regions of low intensity on T2-weighted images, and were drawn directly on the CT images using the MRI scans as a reference, because the absence of free water within fungal hyphae results in marked hypointensity on MRI while calcifications and paramagnetic substances from fungal organisms can contribute to T2 shortening (8). The mucus ROIs were defined as the sinus regions excluding the area with low (fungal regions) or/and high intensity (mucosal thickening region) on T2-weighted images.

Among 86 patients with SFB, the fungal ROIs and the mucus ROIs were determined in 63 patients using MRI. Among 22 patients without calcification, the fungal ROIs and the mucus ROIs were determined in 17 patients using MRI.

Among 97 patients with UCRS, the mucus ROIs were determined in 37 patients with MRI. Among 79 patients without calcification, the mucus ROIs were determined in 29 patients using MRI.

The relative CT number of an individual affected sinus was calculated by dividing the mean CT attenuation value of the ROIs (sinus ROIs, fungal ROIs, or mucus ROIs) by the mean CT attenuation value of the brainstem (15).

Affected sinuses with soft-tissue density within the sinuses were classified as maxillary sinus (MS), ethmoid sinus (ES), frontal sinus (FS), or sphenoid sinus (SS).

On the basis of previous reports (16–19), two categories of bony changes were examined: bone thickening and bone erosion. These changes were analyzed by two independent otolaryngologists who were blinded to the clinical details. Thickening on CT was defined when the cross-section of the bone surrounding the mass showed areas of thickening in three slices compared with the same area on the contralateral side. Erosion on CT was defined as a focal area of cortical loss with sharply defined margins seen on at least three slices. A sinus that was completely filled with material of soft-tissue density was defined as complete opacification, while a sinus with air inclusions was defined as incomplete opacification.

### Statistical Analyses

Continuous and categorical variables are expressed as mean ± standard deviation (SD) and number (%), respectively. Relative CT numbers were compared between SFBs and UCRSs using the Mann–Whitney U test or the Steel-Dwass test. A significance test of non-zero values of the Spearman’s correlation coefficient of the data in Figure 2E was performed.

**Figure 1:**
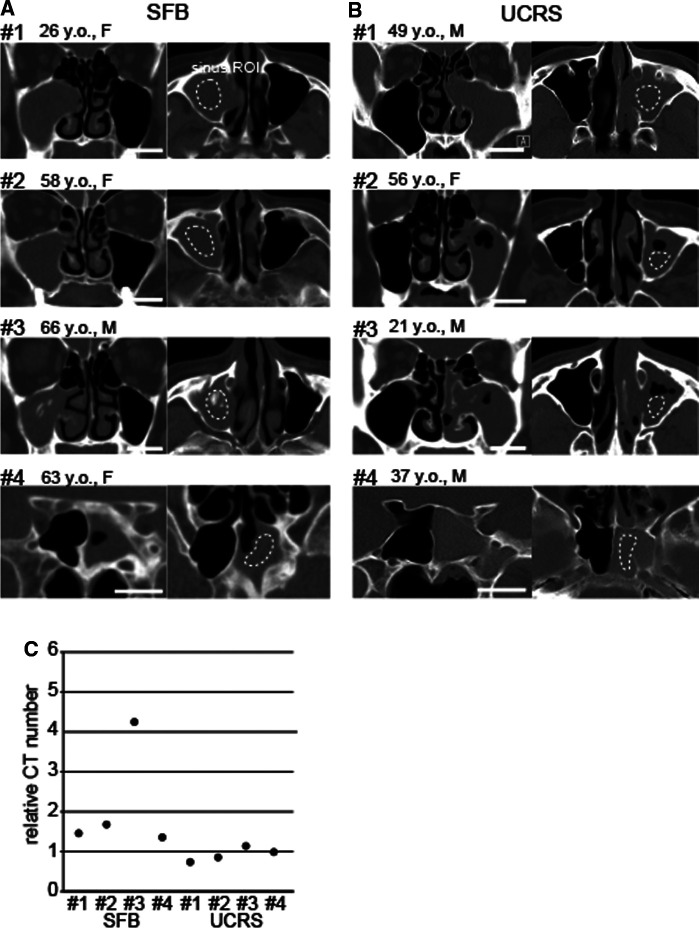
Representative images of sinus fungal ball (SFB) and unilateral nonfungal chronic sinusitis (UCRS), (**A**) CT images of SFB cases (#1–#4). ROIs were defined in the affected sinus (dotted circle), and the brainstem was used as a control area. ROI, region of interest. Scale bar, 20 mm. (**B**) CT images of UCRS cases (#1–#4). ROIs were defined in the affected sinus (dotted circle), and the brainstem was used as a control area. Scale bar, 20 mm. (**C**) Relative CT numbers in SFB and UCRS cases. Each dot represents the relative CT number of an individual case.

**Figure 2:**
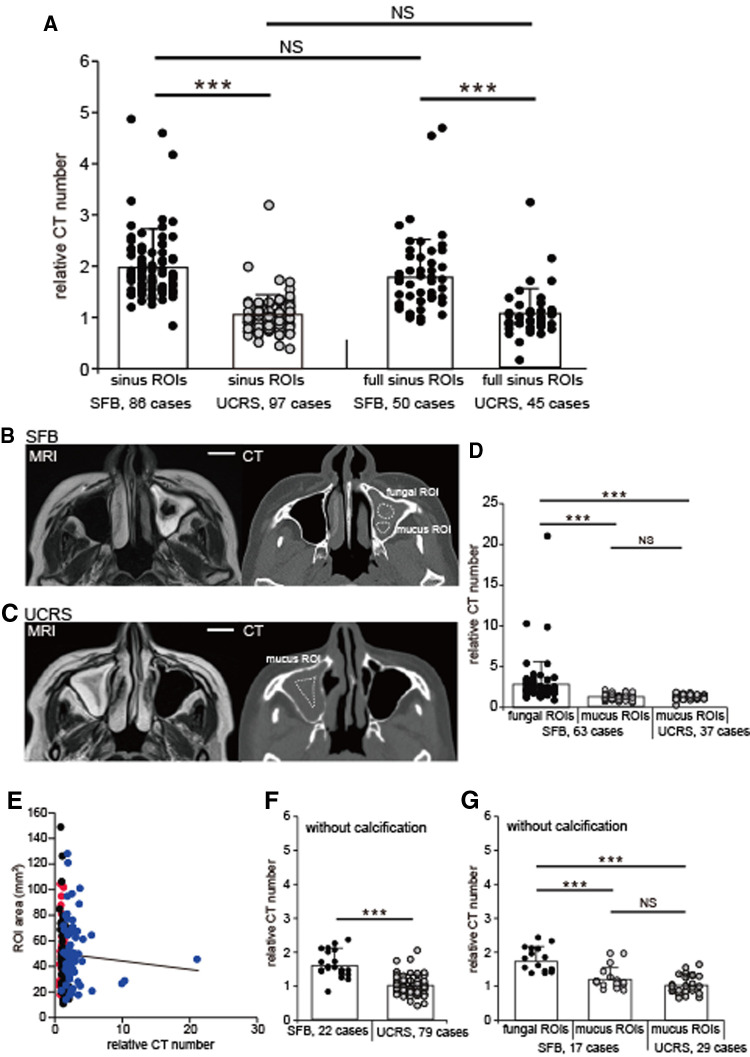
Relative CT numbers of SFB and UCRS cases, (**A**) Relative CT numbers in the sinus and the full sinus ROIs of SFB and UCRS. Each dot represents the relative CT number of an individual SFB or UCRS. All values are mean ± SD. ***p < 0.001; NS, not significant; Mann–Whitney *U* test. (**B**) CT and MRI images of an SFB case. The fungal ROIs were defined as areas with low intensity on T2-weighted images, and were drawn directly on the CT image using the MRI scans as a reference. The mucus ROIs were defined as the sinus ROIs excluding the area with low intensity on T2-weighted images. ROI, region of interest. Scale bar, 20 mm. (**C**) CT and MRI images of an UCRS case. The mucus ROIs were defined as the sinus ROIs excluding the area with high intensity (mucosal thickening region) on T2-weighted images. ROI, region of interest. Scale bar, 20 mm. (**D**) Relative CT numbers in fungal and mucus ROIs. Each dot represents the relative CT number of an individual fungal or mucus ROI in SFBs (63 cases) or mucus ROI in UCRSs (37 cases). All values are mean ± SD. ***p < 0.001, Steel-Dwass test. NS, not significant. (**E**) Correlation between the ROI area and the relative CT numbers. Red, mucus ROIs (UCRS); blue, mucus ROIs (SFB); black, fungal ROIs. (**F**) Relative CT numbers in SFB and UCRS without calcification. Each dot represents the relative CT number of an individual SFB (22 cases) or UCRS (79 cases) without calcification. All values are mean ± SD. Mann–Whitney *U* test. NS, not significant. (**G**) Relative CT numbers in fungal and mucus ROIs without calcification. Each dot represents the relative CT number of an individual fungal or mucus ROI in SFBs (17 cases) or mucus ROI in UCRSs (29 cases) without calcification. All values are mean ± SD. ***p < 0.001, Steel-Dwass test. NS, not significant.

The areas under the receiver operating characteristics curves for relative CT number and age were compared using the χ-square test. Univariable and multivariable logistic regression analyses were performed to identify the factors predictive of disease type (the dependent variable; SFB, 1; UCRS, 0), with the potential predictive factors being age, sex, side, disease duration, nasal symptom (NO, NB, ND, PND, and HD), affected sinus (MS, ES, FS, and SS), relative CT number, sinus ROI size (mm^2^), bony changes (calcification, bone thickening, bone erosion, and complete opacification), BMI, and BI. Associations between predictive and dependent variables are expressed as odds ratios and their 95% confidence intervals (CIs). Variables were included in the multivariable logistic regression analysis if their univariable *p*-value was ≤0.05. A *p*-value of <0.05 was considered statistically significant.

## Results

Of 379 patients who underwent unilateral endoscopic sinus surgery, 209 patients with unilateral chronic sinusitis were identified, and 170 patients were excluded from the analysis (tumor, 108 patients; cyst, 49 patients; antrochoanal polyp, six patients; invasive sinus mycosis with bone destruction, two patients; others, five patients). Of the 209 patients with unilateral chronic sinusitis, 26 were excluded because of the following reasons: CT results were unusable because they were only available on film (nine patients); CT results were for recurrent cases (seven patients); CT scans were performed with intravenous contrast enhancement without an unenhanced scan being acquired (five patients); or the ROI could not be determined because of a narrow range of soft-tissue density (five patients). The remaining 183 patients were included in the study (Table 1) and consisted of 86 patients with SFB and 97 patients with UCRS. Among the 86 patients with SFB, 53 were confirmed to have *Aspergillus*-based SFB according to pathological findings. In the remaining patients, the fungal species could not be identified.

Table 1 lists the demographic and clinical information (age, sex, side, disease duration, nasal symptom, BMI, and BI) of the 183 study patients, and Table 2 summarizes their CT findings (affected sinus, calcification, bone thickness, bone erosion, and complete opacification).

Representative images of four SFB and four UCRS cases are shown in Figures 1A,B. Sinus ROIs were selected from areas within the affected sinus that showed soft-tissue density on non-contrast-enhanced CT. The relative CT numbers for the sinus ROI in each case are shown in Figure 1C (SFB, case #1: 1.5, case #2: 1.7, case #3: 4.2, case #4: 1.3; UCRS, case #1: 0.8, case #2: 0.9, case #3: 1.1, case #4: 1.0).

The relative CT numbers of the affected sinuses are plotted in Figure 2A for all SFBs (86 cases) and all UCRSs (97 cases).

The relative CT numbers of the patients with SFB were significantly higher than those with UCRS (SFBs, 2.0 ± 0.7; UCRS, 1.1 ± 0.4; Figure 2A; Mann–Whitney test, *p* < 0.001). However, the relative CT number may be affected by the ROI settings. Therefore, in addition to the sinus ROIs, the full sinus ROIs were defined and the influence of both ROIs on the relative CT numbers was investigated. In terms of the full sinus ROIs, the relative CT numbers in SFB were significantly higher than those in UCRS (SFBs, 1.9 ± 1.1; UCRS, 1.0 ± 0.5; Mann–Whitney test, *p* < 0.001; Figure 2A). Furthermore, there were no significant differences in the relative CT numbers between the sinus ROIs and the full sinus ROIs in patients with SFBs and UCRSs (sinus ROIs in SFB vs. full sinus ROIs in SFB, *p* = 0.19; sinus ROIs in UCRS vs. full sinus ROIs in UCRS; Mann–Whitney test, *p* = 0.07; Figure 2A). These results suggest that, regardless of the ROI enclosure, the relative CT numbers of the patients with SFB were significantly higher than those with UCRS.

To investigate whether the high relative CT numbers were caused by fungal hyphae inside the sinus, the relative CT numbers were calculated separately for fungal and mucus ROIs (Figures 2B,C). In patients with an SFB, the relative CT numbers of the fungal ROIs were significantly higher than those of the mucus ROIs (fungal ROIs, 2.9 ± 2.9; mucus ROIs, 1.2 ± 0.3; Steel-Dwass test, *p* < 0.001; Figure 2D), and were also significantly higher than the relative CT numbers of mucus ROIs in patients with UCRSs (1.1 ± 0.4; Steel-Dwass test, *p* < 0.001; Figure 2D). However, no significant difference was detected in the relative CT numbers of the mucus ROIs of patients with SFB in comparison with patients with UCRS (Steel-Dwass test, *p* = 0.94; Figure 2D). These results indicate that ROIs with high relative CT numbers coincided with regions where fungal hyphae were likely to be localized. To rule out the possibility that differences in the ROI area affected the relative CT numbers, the correlations between the area of each ROI and relative CT numbers were examined for the sinus ROIs, fungal ROIs, and mucus ROIs. The results showed no significant correlation between the area of each ROI and relative CT numbers (Spearman’s rank correlation coefficient, r^ ^= 0.04; *p* = 0.84; Figure 2E). Therefore, the relative CT numbers depend on the internal properties within the ROIs, not on ROI area.

We next investigated whether the relative CT number is a meaningful parameter for detecting SFB without calcification. The relative CT numbers of the sinus in cases without calcification (SFB, 22 cases; UCRS, 79 cases) are shown in Figure 2F. The relative CT numbers in SFB without calcification were significantly higher than those in UCRS without calcification (SFB, 1.6 ± 0.4; UCRS, 1.0 ± 0.3, Mann–Whitney test, *p* < 0.001; Figure 2F). Furthermore, in patients with SFB, fungal ROIs without calcification showed significantly higher relative CT values than mucus ROIs (fungal ROIs, 1.8 ± 0.4; mucus ROIs, 1.2 ± 0.3; Steel-Dwass test, *p* < 0.001; Figure 2G), as well as significantly higher relative CT values than mucus ROIs in UCRS without calcification (UCRS mucus ROIs, 1.0 ± 0.3; Steel-Dwass test, *p* < 0.001; Figure 2G). However, no significant difference was detected between the relative CT numbers of mucus ROIs in SFB without calcification and mucus ROIs in UCRS without calcification (Steel-Dwass test, *p* = 0.95, Figure 2G). These results suggest that measurement of relative CT numbers could be useful for detecting fungal hyphae, even in cases without calcification.

We next examined the predictive factors for distinguishing between SFB and UCRS by conducting univariable logistic regression analysis to select appropriate parameters, followed by a multivariable logistic regression analysis. In the univariable analysis, age, sex, nasal symptom (ND), affected sinus (MS, ES, FS), relative CT number, and calcification had predictive value for differentiating between SFB and UCRS (age: OR, 1.07, 95% CI, 1.05–1.1; sex: OR, 0.43, 95% CI, 0.24–0.79; ND: OR, 0.39, 95% CI, 0.21–0.71; MS: OR, 0.28, 95% CI, 0.09–0.81; ES: OR, 0.5, 95% CI, 0.28–0.91; FS: OR, 0.24, 95% CI, 0.09–0.59; relative CT number: OR, 33.78, 95% CI, 11.64–98.01; calcification: OR, 12.77, 95% CI, 6.31–25.83; Table 3). In the multivariable logistic regression analysis including the factors of age, sex, nasal symptom (ND), affected sinus (MS, ES, and FS), relative CT number, and calcification, SFB was significantly associated with old age, high relative CT number, and the presence of calcification (age: OR, 1.07, 95% CI, 1.03–1.1, *p* < 0.001; relative CT number: OR, 17.88, 95% CI, 5.4–59.2, *p* < 0.001; calcification: OR, 5.18, 95% CI, 1.87–14.32, *p* = 0.002; Table 3).

**Table 3:** Univariable and multivariable analyses of factors predictive of SFB (SFB, 86 cases; UCRS, 97 cases; *N* = 183).

Variable			Univariable OR (95% CI)	*p*-value	Multivariable OR (95% CI)	*p*-value
Age (years)			1.07 (1.05–1.1)	<0.001	1.07 (1.03–1.1)	<0.001
Sex			0.43 (0.24–0.79)	0.005	0.5 (0.19–1.37)	0.18
Side			1.00 (0.56–1.8)	0.99		
Disease duration (months)			1.00 (0.99–1.01)	0.77		
Nasal symptom	NO		0.66 (0.32–1.39)	0.28		
	NB		1.74 (0.48–6.4)	0.4		
	ND		0.39 (0.21–0.71)	0.002	0.6 (0.22–1.65)	0.32
	PND		0.53 (0.53–1.92)	0.99		
	HD		2.29 (0.87–6.04)	0.09		
Affected sinus	MS		0.28 (0.09–0.81)	0.02	0.51 (0.1–2.48)	0.4
	ES		0.5 (0.28–0.91)	0.02	1.22 (0.41–3.58)	0.72
	FS		0.24 (0.09–0.59)	0.002	0.55 (0.14–2.2)	0.4
	SS		1.69 (0.71–4.04)	0.24		
Relative CT number		SFB, 2.0 ± 0.7	33.78 (11.64–98.01)	<0.001	17.88 (5.4–59.2)	<0.001
		UCRS, 1.1 ± 0.4				
Sinus ROI (mm^2^)		SFB, 170.9 ± 82.5	1.00 (0.99–1.00)	0.13		
		UCRS, 190.7 ± 81.5				
CT	Calcification		12.77 (6.31–25.83)	<0.001	5.18 (1.87–14.32)	0.002
	Bone thickness		0.85 (0.47–1.54)	0.59		
	Bone erosion		1.04 (0.43–2.49)	0.93		
	Complete opacification		1.20 (0.67–2.16)	0.54		
BMI			0.97 (0.89–1.04)	0.39		
BI			1.00 (0.99–1.00)	0.48		

*SFB, sinus fungal ball; UCRS, unilateral nonfungal chronic rhinosinusitis; NO, nasal obstruction; NB, nasal bleeding; ND, nasal discharge; PND, postnasal discharge; HD, headache; MS, maxillary sinus; ES, ethmoidal sinus; FS, frontal sinus; SS, sphenoid sinus; ROI, region of interest; BMI, body mass index; BI, Brinkman index; OR, odds ratio; CI, confidence interval*.

We further examined the predictive factors for distinguishing between SFB and UCRS in cases without calcification by conducting univariable logistic regression analysis to select appropriate parameters, followed by a multivariable logistic regression analysis. In the univariable analysis, age, sex, affected sinus (MS and SS), and relative CT number had predictive value for differentiating between SFB and UCRS (age: OR, 1.06, 95% CI, 1.02–1.11; sex: OR, 0.37, 95% CI, 0.14–0.98; MS: OR, 0.09, 95% CI, 0.02–0.35; SS: OR, 4.14, 95% CI, 1.3–13.18; relative CT number: OR, 15.58, 95% CI, 4.28–56.67; Table 4). In the multivariable logistic regression analysis including the factors of age, sex, affected sinus (MS and SS), and relative CT number, SFB was significantly associated with old age and high relative CT numbers (age: OR, 1.08, 95% CI, 1.02–1.14, *p* = 0.004; relative CT number: OR, 17.58, 95% CI, 3.66–84.34, *p* < 0.001; Table 4).

**Table 4:** Univariable and multivariable analyses of factors predictive of SFB without calcification (SFB, 22 cases; UCRS, 79 cases; *N* = 101).

Variable			Univariable OR (95% CI)	*p*-value	Multivariable OR (95% CI)	*p*-value
Age (years)	SFB, 57.7 ± 13.3		1.06 (1.02–1.11)	0.003	1.08 (1.02–1.14)	0.004
	UCRS, 45.3 ± 15.8					
Sex	SFB	M, 8 (36%)	0.37 (0.14–0.98)	0.04	0.34 (0.09–1.26)	0.11
		F, 14 (64%)				
	UCRS	M, 48 (61%)				
		F, 31 (39%)				
Side	SFB	R, 10 (45%)	0.7 (0.27–1.8)	0.46		
		L, 12 (55%)				
	UCRS	R, 43 (54%)				
		L, 36 (46%)				
Disease duration (months)	SFB, 14.5 ± 31.35		1.00 (0.98–1.02)	0.93		
	UCRS, 13.7 ± 22					
Nasal symptom	NO	SFB, 3 (14%)	2.53 (0.4–16.2)	0.33		
		UCRS, 18 (23%)				
	NB	SFB, 12 (55%)	1.74 (0.48–6.4)	0.33		
		UCRS, 3 (4%)				
	ND	SFB, 7 (32%)	0.41 (0.15–1.12)	0.08		
		UCRS, 42 (53%)				
	PND	SFB, 10 (45%)	1.8 (0.69–4.72)	0.23		
		UCRS, 25 (32%)				
	HD	SFB, 1 (5%)	0.89 (0.09–8.42)	0.92		
		UCRS, 4 (5%)				
Affected sinus	MS	SFB, 14 (64%)	0.09 (0.02–0.35)	<0.001	0.36 (0.04–3.72)	0.39
		UCRS, 75 (95%)				
	ES	SFB, 11 (50%)	0.76 (0.29–1.95)	0.56		
		UCRS, 45 (57%)				
	FS	SFB, 4 (18%)	0.58 (0.18–1.89)	0.36		
		UCRS, 22 (28%)				
	SS	SFB, 7 (32%)	4.14 (1.3–13.18)	0.02	1.58 (0.16–15.84)	0.7
		UCRS, 8 (10%)				
Relative CT number		SFB, 1.61 ± 0.52	15.58 (4.28–56.67)	<0.001	17.58 (3.66–84.34)	<0.001
		UCRS, 1.04 ± 0.27				
Sinus ROI (mm^2^)		SFB, 167.7 ± 87.1	0.99 (0.99–1.00)	0.18		
		UCRS, 190.9 ± 77.4				
CT	Bone thickness	SFB, 6 (27%)	0.55 (0.19–1.56)	0.26		
		UCRS, 32 (41%)				
	Bone erosion	SFB, 0 (0%)	Nd	nd		
		UCRS, 5 (6%)				
	Complete opacification	SFB, 14 (64%)	0.39 (0.52–0.39)	0.51		
		UCRS, 44 (56%)				
BMI		SFB, 23.0 ± 4.3	0.96 (0.84–1.09)	0.48		
		UCRS, 23.8 ± 3.7				
BI		SFB, 179.2 ± 267.7	1.00 (0.99–1.00)	0.26		
		UCRS, 88.6 ± 225.7				

*SFB, sinus fungal ball; UCRS, unilateral nonfungal chronic rhinosinusitis; M, male; F, female; R, right; L, left; NO, nasal obstruction; NB, nasal bleeding; ND, nasal discharge; PND, postnasal discharge; HD, headache; MS, maxillary sinus; ES, ethmoidal sinus; FS, frontal sinus; SS, sphenoid sinus; ROI, region of interest; BMI, body mass index; BI, brinkman index; OR, odds ratio; CI, confidence interval; nd, not determined*.

**Figure 3:**
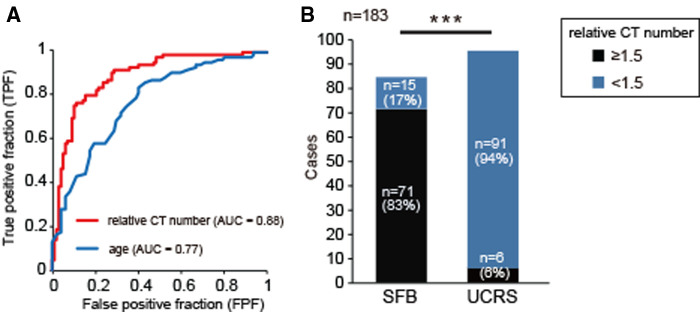
Receiver operator characteristics (ROC) curve analysis for relative CT number and age, (**A**) ROC curves for application of the classification modeling to the dataset. The curve was created by plotting the true positive fraction (TPF, y-axis; the sensitivity) against the false positive fraction (FPF, x-axis; the specificity) at various threshold settings. The red line indicates the ROC curve for relative CT number, and the blue line indicates the curve for age. The area under the ROC curve for relative CT number was significantly greater than that for age (age vs. relative CT number, 95% CI, 0.03–0.21; χ-square test, *p* = 0.008). (**B**) Comparison of relative CT numbers between SFB and UCRS. A relative CT number greater than or equal to 1.5 was significantly associated with the detection of SFB. Chi-square test, *n* = 183, ***p < 0.001.

Receiver operating characteristics analysis was performed to evaluate the accuracy of a statistical model. The areas under the receiver operating characteristic curve (AUC) for relative CT number and age were 0.88 (relative CT number, 95% CI, 0.84–0.94; *p* < 0.001) and 0.77 (age, 95% CI, 0.7–0.84; *p* < 0.001), respectively. The AUC for relative CT number was significantly larger than that for aging (age vs. relative CT number, 95% CI, 0.03–0.21; χ-square test, *p* = 0.008, Figure 3A). These results suggest that the measurement of relative CT number is a more accurate diagnostic method for detecting fungal hyphae than patient age. From the AUC, the optimum relative CT number cut-off value for determining SFB was calculated as 1.5 (AUC, 0.88; OR, 22.6; sensitivity, 70%; specificity, 91%; Figure 3A); most patients with a relative CT number of less than 1.5 had UCRS, while most of those with a value greater than or equal to 1.5 had SFB (Figure 3B, *χ^2^* test, *p* < 0.001). These results suggest that the relative CT number is a powerful and objective diagnostic tool for differentiating SFB from UCRS.

## Discussion

SFB is an extramucosal intrasinusal mycosis, which usually occurs as a unilateral lesion in immunocompetent people (20). Univariate analyses have revealed SFB to be more common in elderly and female patients (21–23). Although both factors were significant predictors in our univariable analysis, it is noteworthy that only old age was a significant factor in the multivariable analysis, and old age can therefore be considered a patient characteristic for differentiating SFB from UCRS.

The preoperative diagnosis of unilateral sinusitis can usually be made according to the clinical symptomatology and radiological findings. Nasal obstruction and unilateral nasal discharge are the most frequent complaints (24), although the nasal symptoms of SFB are nonspecific and similar to those of chronic sinusitis because they depend strongly on the location and subsequent involvement of surrounding structures (23, 25, 26).

Various radiological diagnostic signs for SFB are helpful in assessment and clinical decision making. Previous studies have reported well-verified CT features of SFB, including the presence of calcification, complete opacification, and bone thickening (27). In the present study, no bony changes on CT were found to be significant factors in the univariate analysis. The sinus wall is not a static structure: it responds to stimuli such as mechanical stress and inflammation by altering and repairing its structure through a process referred to as remodeling (28). Because most fungal hyphae have little or no invasive ability, it has been speculated that bony changes in SFB are related to the duration of active inflammation within the affected sinus (25, 28). In our analysis, we did not find a significant difference in disease duration between SFB and UCRS, which may be why we did not find any significant difference in the frequency of bony changes between the two diseases.

Our multivariable analysis revealed sinus calcification on CT to be a factor significantly associated with SFB. This finding could represent the presence of heavy metals (iron, zinc, and manganese) and calcium within fungal hyphae, and is considered to be a reliable diagnostic feature of SFB (29, 30). However, the presence of calcified fungal hyphae might not be confirmed in some cases. *Aspergillus*, which is the organism most commonly involved in sinus SFBs (31), stores metal ions such as zinc in the intracellular vacuole storage system, with the zinc ion known to be an essential nutrient for the organism (32). However, down regulation of the genes related to zinc storage occasionally occurs, and under such conditions, the zinc concentration in the vacuole decreases, which may be reflected by the absence of calcification (33). In agreement with previous reports (1, 2, 10–12), we could not detect calcification within the affected sinus in 26% of the patients with SFB. Other than sinus calcification, objective CT-based indicators strongly predicting the presence of fungal hyphae have not been established.

On MRI, a marked hypointensity on T2-weighted imaging (T2WI) is a significant predictor of SFB (6). However, in clinical practice, marked hypointensity lesions on T2WI are also observed in intra-sinonasal desiccated secretions, air, and even acute clotted hemorrhage (2, 34). Furthermore, because of the high cost of MRI, MRI examinations are not usually performed on all patients. Therefore, it would be clinically meaningful to find a new CT-based diagnostic indicator to detect fungal hyphae.

The CT attenuation values assigned to a voxel represent the average linear attenuation coefficient of that voxel relative to water. They are primarily influenced by the chemical composition of the tissue and organ (e.g., −1,000 HU for air, 0 HU for water, and 1,000 HU for bone) (14). However, CT attenuation values can be affected by the settings used for image reconstruction. In previous studies, we established a method for measuring relative CT numbers in relation to brainstem values, and reported the usefulness of the measurement for the differential diagnosis of individual tumors (15, 19, 35). The measurement of relative CT numbers can be applied to inflammatory sinus diseases, and the evaluation of sinus ROIs can reveal the characteristics of the internal properties of sinuses. In SFB, relative CT numbers could be used to evaluate the concentration of heavy metals inside the affected sinus, which would help to diagnose the presence of fungal hyphae.

Our diagnostic process for SFB on CT involves measuring the relative CT number and checking for unilateral opacification of the sinus with calcification. If the relative CT number is greater than 1.5, or if sinus calcification is present, then SFB can be suspected. Indeed, in elderly patients without sinus calcification, high relative CT numbers may be the only basis for strongly suspecting SFB.

The ability to define relative CT numbers in individual sinus diseases may provide meaningful information for differentiating neoplastic from inflammatory lesions within the nasal cavity. For example, the mean relative CT number of inverted papilloma is reported to be 1.3 (35), whereas that of SFB is considerably higher at 2.0. If specific relative CT numbers can be defined for various sinus diseases, it might be possible to broadly classify sinus diseases according to the differences in these relative CT numbers.

The current study has several limitations. First, it is of a retrospective cohort design and is subject to the inherent biases that come with retrospective studies. Second, our study was performed on a limited number of SFB cases, particularly SFB without calcification, and all patients presented at a single institution. Therefore, the generalizability of our findings to other settings is unclear. Third, further studies are needed to determine cut-off values for relative CT numbers if they are to be used for differentiation of the diseases in larger numbers of patients. Nevertheless, this is the first study to show that relative CT numbers can provide useful information for the radiological diagnosis of SFB.

## Conclusion

In the present study, we used univariable and multivariable regression analyses to determine the predictive factors for differentiating between SFB and UCRS. The relative CT numbers of SFB were significantly higher than those of UCRS, not only in the analysis of the fungal ROIs but also in the analysis of sinus ROIs (ROIs encompassing the fungus and the mucus). In the multivariable analysis, age, relative CT number, and calcification had predictive value for distinguishing SFB from UCRS. In patients not showing calcification, age and a relative CT number >1.5 were significantly associated with SFB, with relative CT number showing a significantly larger area under the receiver operating characteristics curve than patient age. These results suggest that the measurement of relative CT number can be a useful non-invasive diagnostic tool for the differentiation of SFB from UCRS.

## Data Availability

The raw data supporting the conclusions of this article will be made available by the authors, without undue reservation.
